# A topological transition by confinement of a phase separating system with radial quenching

**DOI:** 10.1038/s41598-019-52037-4

**Published:** 2019-10-31

**Authors:** Tsuyoshi Tsukada, Rei Kurita

**Affiliations:** Department of Physics, Tokyo Metropolitan University, 1-1 Minamioosawa, Hachiouji-shi, Tokyo, 192-0397 Japan

**Keywords:** Phase transitions and critical phenomena, Coarse-grained models

## Abstract

Physicochemical systems are strongly modified by spatial confinement; the effect is more pronounced the stronger the confinement is, making its influence particularly important nanotechnology applications. For example, a critical point of a phase transition is shifted by a finite size effect; structure can be changed through wetting to a container wall. Recently, it has been shown that pattern formation during a phase separation is changed when a system is heterogeneously quenched instead of homogeneously. Flux becomes anisotropic due to a heterogeneous temperature field; this suggests that the mechanism behind heterogeneous quenching is different from that of homogeneous quenching. Here, we numerically study the confinement effect for heterogeneously quenched systems. We find that the pattern formed by the phase separation undergoes a topological change with stronger confinement i.e. when the height of a simulation box is varied, transforming from a one-dimensional layered pattern to a two-dimensional pattern. We show that the transition is induced by suppression of the heterogeneous flux by spatial confinement. Systems with heterogeneous flux are ubiquitous; the effect is expected to be relevant to a wide variety of non-equilibrium processes under the action of spatial confinement.

## Introduction

Confining a system to a container of finite size is known to affect both static and dynamic phenomena. This includes critical phenomena, phase transitions^1–3^, glass transitions and crystallization^4,5^. Understanding the effect is important both for elucidating the mechanisms behind non-equilibrium phenomena and for nanotechnology applications^6^, where spatial confinement might extend to length scales approaching the size of molecules. An example is a critical point: it is known that the critical point can be shifted or smoothed by spatial confinement when the size of the container is close to a correlation length. This so-called finite size effect has been widely reported^1^. In addition, the effect of the wall itself may become significant^4,5,7^; when one component of a binary system has greater affinity to a wall, the local fraction of the component near the wall increases^8–11^. The smaller the container, the larger the surface-to-volume ratio, leads to a more pronounced effect due to wetting. It is also known that the mobility of a fluid is modified by the smoothness of a wall^7^; the mobility is enhanced if the wall is smooth and reduced near the rough wall. Even the shape of the container may affect phase transitions e.g. crystallization. In a carbon nanotube, the crystalline structure of water has been recently shown to be distinct from that in bulk^12^.

The effect of the confinement has been studied for a wide range of non-equilibrium phenomena such as phase separations, glass transitions etc., however it is yet to be considered for systems phase separating under an inhomogeneous quench. The patterns which emerge from inhomogeneous quenching in bulk have been found to be different from those formed via homogeneous quenching^13–23^. In particular, the phase separation with a quenching front propagating in a single direction i.e. a directional quenching method was proposed some decades ago^13^. A front separating quenched and non-quenched regions propagates at a constant velocity $V$; this parameter has been shown to control the formation of a series of metastable patterns like columns and lamella^13–23^. The dynamics of phase separation with inhomogeneous quenching is also different, distinct from the model B universality class^24^, despite the final temperature being the same for both homogeneous and inhomogeneous quenching.

Recently, Tsukada and Kurita reported pattern formation by radial quenching (RQ) of a binary fluid with realistic boundary conditions in three dimensions. Radial quenching refers to the propagation of the quenching front, which propagates in a radial direction from a pre-defined center. In the study, RQ was applied to the bottom surface of a container, and the temperature allowed to evolve via thermal diffusion away from the bottom surface i.e. in the $z$ direction^25^. This is analogous to an actual experiment, where one would control the temperature of the bottom surface of a sample using a heating or cooling stage. It was found that a layered pattern was observed in the $z$ direction, topologically distinct from the patterns seen for RQ in two dimensions using numerical simulations. During radial quenching via heating/cooling of the bottom surface, the flux of the majority component becomes anisotropic due to an inhomogeneous temperature field. The flux in the $z$ direction is larger than the flux in the $xy$ plane, as a result, layers are formed. This suggests that anisotropic flux plays an important role for pattern formation in inhomogeneous quenched systems. Note that this topological change is brought about by a change in dimensionality^18,25^: this strongly suggests that spatial confinement may have a similar effect i.e. affect anisotropic flux. Thus, the objective of this paper is to study the effect of confinement on a system with inhomogeneous quenching. We performed numerical simulations with radial quenching for systems with different simulation box heights, giving rise to topologically distinct patterns (see Methods section). We thus aim to clarify the mechanism behind the formation of the different patterns and show specifically how the spatial confinement suppresses the formation of an anisotropic flux.

## Results

### Pattern diagram

Firstly, we briefly introduce our simulation method. The inhomogeneous quenching was applied to the bottom surface, and that evolution of the temperature was governed by conduction. For an inhomogeneous temperature field, a modified Cahn-Hilliard-Cook equation was proposed^26^. The system studied here is initiated with a temperature $T(\overrightarrow{r})={T}_{0}(\, > \,{T}_{c})$ at all positions $\overrightarrow{r}$. ${T}_{c}$ is a critical temperature. We normalize the length and the time using the correlation length ${\xi }_{0}$ and the characteristic time ${\tau }_{0}$, respectively. Next, we define a normalized temperature $\varepsilon (\overrightarrow{r},t)$ as $\varepsilon (\overrightarrow{r},t)=(T(\overrightarrow{r},t)-{T}_{c})/({T}_{0}-{T}_{c})$. We thus obtain normalized equations as shown below, and normalized equations are given below,

1 $$\frac{\partial \varphi }{\partial t}={\nabla }^{2}[\epsilon \varphi +{\varphi }^{3}-{\nabla }^{2}\varphi ]+\theta $$

2 $$\frac{\partial \epsilon }{\partial t}=Le{\nabla }^{2}\epsilon ,$$

where *θ* and $Le(\,=\,K/L)$ are a random flux and the Lewis number, respectively. We set the mesh size to 1 and the time step to 0.01. The simulation box size was *x* : *y* : *z* = 128 : 128 : *h*; *h* is a positive integer and the key parameter here for investigating the effect of confinement. The mean concentration is set to $\bar{\varphi }$ = 0.1; note that this denotes an asymmetric composition. Having set up the simulation in this manner, we start radially quenching outwards from a center on the bottom surface with a constant velocity ${V}_{xy}$ at $t=0$. Details for our simulation method is described at the Methods section.

**Figure 1:**
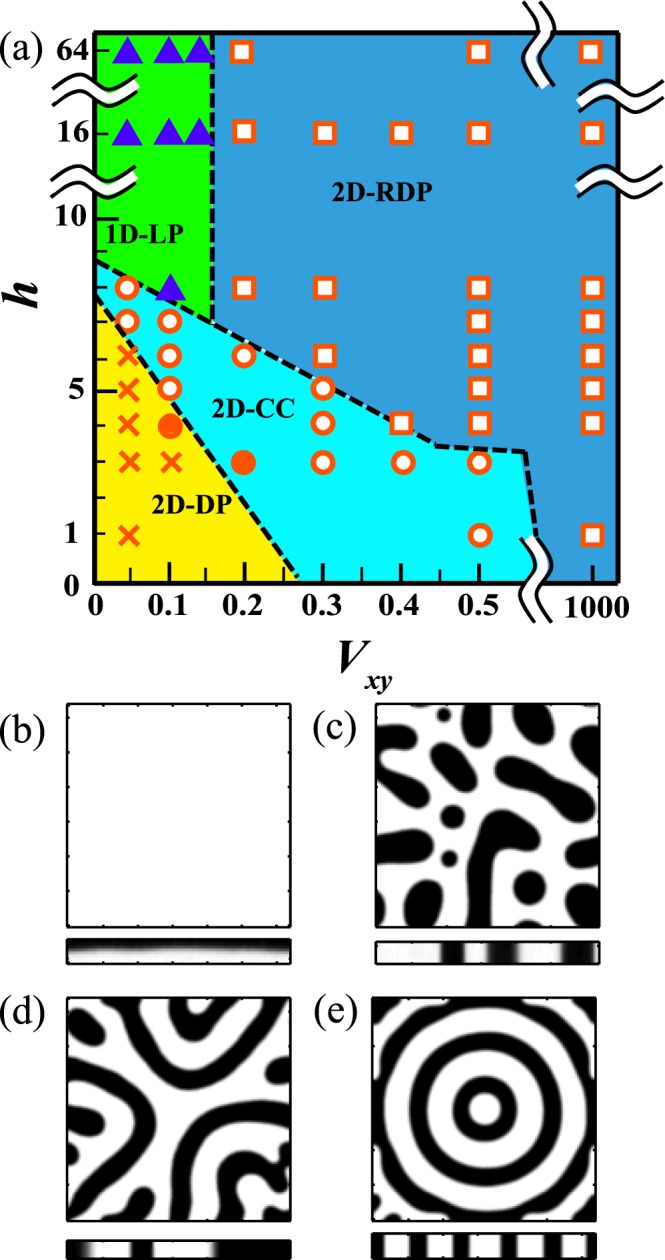
(**a**) Different patterns as a function of ${V}_{xy}$ and $h$. Symbols represent simulated points. Squares correspond to a 2 dimensional random droplet pattern (2D-RDP), similar to what is seen in homogeneous quenching; filled triangles correspond to 1 dimensional layer pattern (1D-LP), which can be observed at lower ${V}_{xy}$ and larger $h$; circles correspond to 2 dimensional concentric circles (2D-CC); crosses correspond to 2 dimensional dendritic pattern (2D-DP). 2D-CC and 2D-DP are formed at smaller $h$. (**b**–**e**) Cross sections of each pattern at *t* = 1000 and at *z* = 0 (upper) and at *y* = 64 (lower). (**b**) Cross sections of 1D-LP at (${V}_{xy}$, $h$) = (0.1, 8). (**c**) Cross sections of 2D-RDP at (${V}_{xy}$, $h$) = (1000, 3). (**d**) Cross sections of 2D-DP at (${V}_{xy}$, $h$) = (0.1, 3). (**e**) Cross sections of 2D-CC at (${V}_{xy}$, $h$) = (0.1, 5).

Then we investigate the pattern formed during phase separation for different $h$ and ${V}_{xy}$. Figure 1(a) shows the patterns found at later stages of the phase separation process. Symbols in Fig. 1(a) are simulated points and the dotted lines are guides for the eye denoting boundaries between each regime. Figure 1(b–e) shows the concentration fields $\varphi$ for typical patterns. The upper images of Fig. 1(b–e) show cross sections of $\varphi$ at *z* = 0, while the lower images show cross sections at *y* = 64.

We briefly describe each regime in the diagram. When *h* = 64 and ${V}_{xy} < 0.2$, a layered pattern is formed in the *z* direction; this is denoted as a filled triangle in Fig. 1(a). The observation of layers is consistent with previous work^25^. We note that the dimensionality of the pattern can be reflected in the naming of each pattern. Since $\varphi$ of a layered pattern may be expressed as $\varphi (z)$ as shown in Fig. 1(b), we call this a 1 dimensional layer pattern (1D-LP). When *h* = 64 and for ${V}_{xy} > 0.2$, both phases percolate in the *z* direction, but the positions of both phases are random in the *xy* plane; this is denoted by a square symbol in Fig. 1(a). The pattern is shown in Fig. 1(c); we call this a 2 dimensional random droplet pattern (2D-RDP). Note points denoted by a cross on the phase diagram; for these, a dendritic pattern is observed in the *xy* plane, while the pattern percolates in the *z* direction (Fig. 1(d)). The pattern is consistent with simulations in two dimensions with radial quenching^18^. We call this a 2 dimensional dendritic pattern (2D-DP). Finally, we also find concentric circles (Fig. 1(e)), denoted by circles on the diagram, seen for small *h* and a mid-range ${V}_{xy}$. Since the pattern can be determined by ${r}_{c}$ and $r$, where ${r}_{c}$ is the position of the predefined center and $r$ is a distance from ${r}_{c}$, we call these 2 dimensional concentric circles (2D-CC). The filled circles correspond to a coexistence pattern of 2D-DP and 2D-CC.

Here, note that a transition between 1D and 2D patterns is observed not only for different ${V}_{xy}$, but also $h$. It is clear that the effect of confinement plays an important role for determining the boundary between patterns with distinct topological characteristics.

### 2D-RDP regime

**Figure 2:**
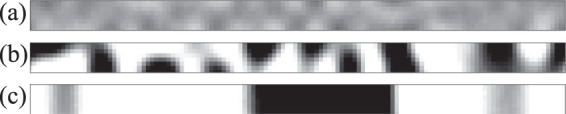
Time evolution of concentration at *y* = 64 when *V*_*xy*_ = 1000 and *h* = 8. *t* = (**a**) 10, (**b**) 50, (**c**) 700. Spherical droplets appear at an early stage, and droplets coarsen over time. Since the droplets percolate in the *z* direction, the pattern becomes a disk at later stages.

**Figure 3:**
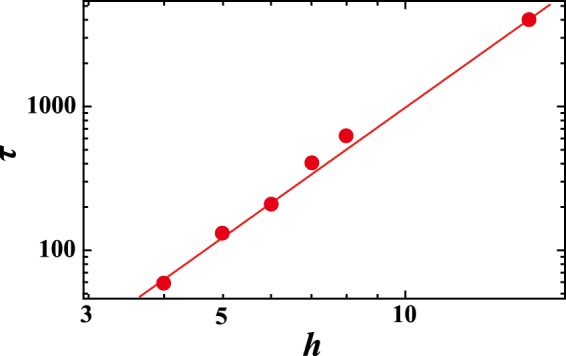
Time $\tau$ when the random droplets percolate in the $z$ direction for the first time, as a function of $h$. The solid line corresponds to $\tau =0.98\,{h}^{3}$.

Here, we show how 2D-RDP evolves over time. Figure 2 shows the time evolution of the pattern at *h* = 8 and *V*_*xy*_ = 100. Firstly, the system is almost homogeneously quenched since ${V}_{xy}$ is quite fast. Thermal fluctuations then grow over time since the temperature is in the spinodal decomposition region (Fig. 2(a)). After the early stages of phase separation, a minority phase appears as droplets (Fig. 2(b)). The droplets become larger as coarsening takes place to decrease interfacial energy. When the size of a droplet exceeds $h$, the minority phase percolates in the $z$ direction. Finally, the interface becomes flat in the $xz$ plane to further decrease the interfacial energy, and the pattern becomes 2 dimensional (Fig. 2(c)). Here, we also compute $\tau$, the time it takes for the minority phase to percolate in the $z$ direction for the first time. It is found that $\tau \propto {h}^{3}$, as shown in Fig. 3. This is consistent with $\xi \sim {t}^{\mathrm{1/3}}$ for homogeneous quenching, where $\xi$ is a typical length of the pattern. We also find that there is no anomaly for $\tau$ with changing $h$. This suggests that the mechanism of the pattern formation is essentially independent of $h$.

### Transition between 1D-LP and 2D-RDP

**Figure 4:**
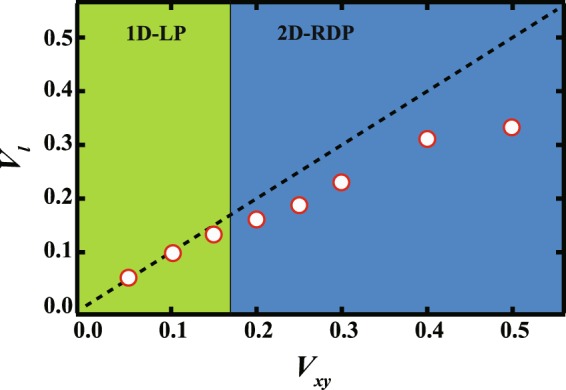
*V*_*xy*_ dependence of the growth velocity *V*_*l*_ of the bottom layer in the *xy* plane when *h* = 16. The dashed line shows when ${V}_{l}={V}_{xy}$; the dotted red line represents the boundary between 2D-RDP for larger *V*_*xy*_ and 1D-LP for smaller ${V}_{xy}$. When ${V}_{xy}\sim {V}_{l}$, the layer grows with the spread of the quenching domain, forming a 1D-LP. If ${V}_{l}$ is smaller than ${V}_{xy}$, droplets appear randomly.

Next, we focus on the change in the pattern from 2D-RDP to 1D-LP with decreasing ${V}_{xy}$ when $8\le h\le 64$. The dynamics of this regime was reported in ref.^25^; here, we briefly review the mechanism behind 1D-LP formation. When ${V}_{xy}$ is small, the majority phase appears at the center of the quenched region on the bottom surface. Due to the difference in the temperature gradient in the $z$ and $xy$ directions, flux in the $z$ direction becomes dominant during the phase separation. The majority phase then grows in the $xy$ plane while the minority phase appears on the top surface of the bottom phase, eventually leading to a 1 dimensional layer pattern. Here, we compute the growth velocity parallel to the $xy$ plane ${V}_{l}$ with changing ${V}_{xy}$ in the early stages of radial quenching. Figure 4 shows ${V}_{l}$ as a function of ${V}_{xy}$ at *h* = 16; the dashed line corresponds to ${V}_{l}={V}_{xy}$. When ${V}_{xy}$ is less than 0.2, it is found that ${V}_{l}\approx {V}_{xy}$, while ${V}_{l} < {V}_{xy}$ for ${V}_{xy} > 0.2$. We also find that the point where ${V}_{l}$ deviates from ${V}_{l}={V}_{xy}$ is consistent with the boundary between 1D-LP and 2D-RDP (${V}_{xy}\sim 0.2$ i.e. the vertical dashed line in Fig. 4). Looking at the bottom region, the bottom phase grows with the expansion of the quenched region for smaller ${V}_{xy}$, while it cannot keep up with the quenched region when ${V}_{l} < {V}_{xy}$; thus, there is space in the bulk between the edge of the quenched region and the edge of the bottom phase. This leads to random droplets forming far from the center of the quenched region. Note that the relationship between ${V}_{l}$ and ${V}_{xy}$ is independent of $h$ for $8\le h\le 64$; this is consistent with the fact that the boundary between 1D-LP and 2D-RDP is independent of $h$, as shown in Fig. 1(a).

### Transition between 1D-LP and 2D-CC

**Figure 5:**
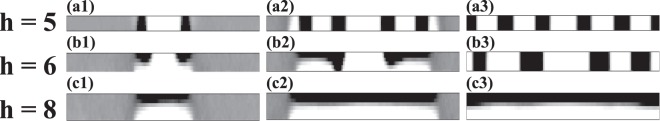
Time evolution at an early stage for fixed ${V}_{xy}=0.1$ (**a**) at *h* = 5 and *t* = (a1) 200, (a2) 450, and (a3) 1000. (**b**) *h* = 6 and *t* = (b1) 200, (b2) 450, and (b3) 1250. (**c**) *h* = 8 and *t* = (c1) 200, (c2) 500, and (c3) 1000. When *h* = 5, droplets are seen to appear in the $xy$ plane at an early stage; on the other hand, when *h* = 8, a layer pattern is formed in the *z* direction in stead. This early pattern formation strongly affects the pattern at later stages. When *h* = 6, the majority phase percolates at the center followed by another adjacent layer in the $z$ direction. Finally, this layer coarsens into the layer in the $xy$ plane.

Next, we investigate the boundary between 1D-LP and 2D-CC. We find that the pattern changes from 1D-LP to 2D-CC with decreasing simulation box height $h$ for ${V}_{xy} < 0.15$. The patterns are topologically different, suggesting that the mechanism behind pattern formation is essentially different. Figure 5 shows early time evolution when ${V}_{xy}=0.1$ for *h* = (a) 5, (b) 6, and (c) 8. When *h* = 5, the majority phase at the center percolates in the *z* direction at the beginning; the minority phase subsequently emerges next to the percolated majority phase as shown in Fig. 5(a1–a3). Finally, the 2D-CC pattern is formed [also see Fig. 1(e)]. When *h* = 6, near the boundary between 1D-LP and 2D-CC, the formation process is dynamically distinct from when *h* = 5, although we still see a 2D-CC pattern at a later stage. Firstly, just like when *h* = 5, the majority phase emerges at the center and percolates in *z*. After this, a 1D-LP like structure appears next to the majority phase as shown in Fig. 5(b1). It is important to note that the next body of minority phase does not percolate in the *z* direction. Since the time evolution strongly depends on local features of the pattern, the layer grows in the $xy$ direction as shown in Fig. 5(b2). At the same time, the minority phase coarsens toward the bottom surface parallel to the percolated majority phase at the core. Finally, when *t* = 450, the minority phase percolates in the $z$ direction. Furthermore, the following volume of majority phase also coarsens toward the top surface around the percolated minority phase. This process repeats, and the initial 1D-LP like structure finally transforms into a 2D-CC structure. When *h* = 8 (Fig. 5(c1)), phase separation occurs at the center in the $z$ direction while the layers grow in the $xy$ plane [Fig. 5(c2)], forming layers. The pattern is stable over time, [Fig. 5(c3)] and is notably different from when *h* = 6.

**Figure 6:**
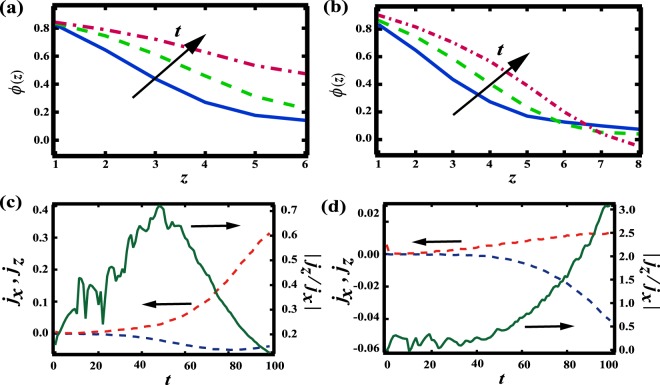
Time evolution of $\varphi$ at the center (*x*, *y*) = (64, 64) as a function of $z$ when *V*_*xy*_ = 0.1 and (**a**) *h* = 6, (**b**) *h* = 8. The solid line, dashed line and dot-dash line correspond to *t* = 50, 75, and 100, respectively. When *h* = 6, the minimum of the concentration increases; when *h* = 8, the minimum decreases. We also show the time evolutions of the flux in the $xy$ plane ${j}_{x}$ (dashed red line), the flux in $z$ direction ${j}_{z}$ at the center on the top surface (dashed blue line), and $|{j}_{z}/{j}_{x}|$ (solid green line) at (**c**) *h* = 6 and (**d**) *h* = 8. It is found that ${j}_{z}$ is dominant at *h* = 8, while ${j}_{x}$ is dominant for $h\le 6$.

We may also investigate the appearance of the majority phase at the beginning of the radial quench in more detail. Figure 6 shows the time evolution of $\varphi$ as a function of $z$ at the center until *t* = 100 for (a) *h* = 6 and (b) *h* = 8. When *h* = 6, we find that $\varphi$ increases for any *z* and the majority phase percolates at the center. On the other hand, when *h* = 8, $\varphi$ increases at small *z* while decreasing for larger $z$. This decrease of $\varphi$ when *h* = 8 triggers the phase separation in the *z* direction. Here we compute the flux $\overrightarrow{j}$ at the center on the top surface using $\overrightarrow{j}=\nabla [\epsilon \varphi +{\varphi }^{3}-{\nabla }^{2}\varphi ]$. Figure 6(c,d) show the flux from the $x$ direction ${j}_{x}$ (red dashed line), the flux from the $z$ direction ${j}_{z}$ (blue dashed line) and $|{j}_{z}/{j}_{x}|$ (green solid line) for *h* = 6 and *h* = 8, respectively. We find that ${j}_{x}$ is dominant for $h < 6$ and then $\varphi$ increases for any $z$. It suggests that ${j}_{z}$ is suppressed by the confinement before ${j}_{z}$ becomes dominant. Meanwhile, ${j}_{z}$ overcomes ${j}_{x}$ for $t > 60$ for $h > 8$, thus $\varphi$ decreases for larger $z$. Thus, the direction of the flux changes due to spatial confinement and it induces a topological transition.

**Figure 7:**
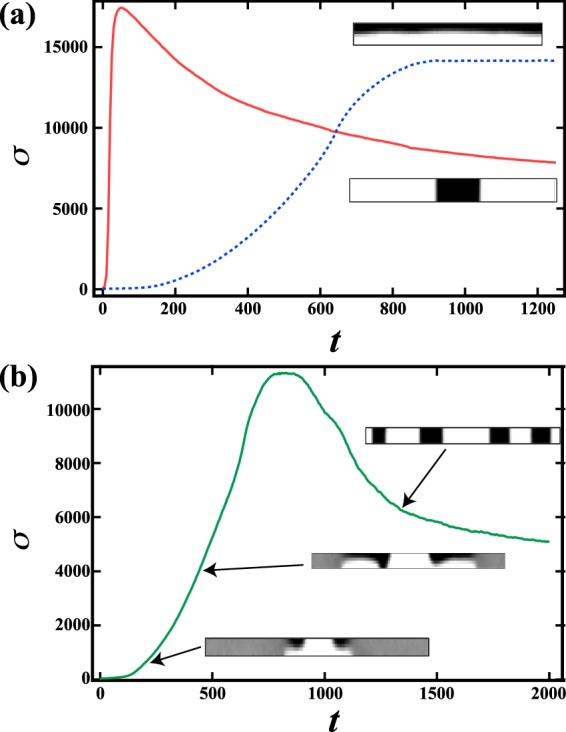
(**a**) Time evolution of interfacial energy $\sigma$ when *h* = 8. The solid line corresponds to when *V*_*xy*_ = 1000; the dashed line corresponds to when *V*_*xy*_ = 0.1. The upper inset corresponds to the cross section at *y* = 64 when *V*_*xy*_ = 0.1 and *t* = 1000; the lower inset corresponds to the cross section at *y* = 64 when *V*_*xy*_ = 1000 and *t* = 1000. It is found that the interfacial energy in the 2 dimensional pattern is smaller than that in the 1 dimensional layer pattern. (**b**) *σ* when *h* = 6 and *V*_*xy*_ = 0.1. The insets are the cross sections at *y* = 64, *t* = 200 (lower), 450 (middle), and 1250 (upper). Firstly, *σ* is seen to increase significantly due to the formation of a layer pattern. Subsequently, *σ* decreases due to transformation of the layer to a 2 dimensional percolated pattern.

The coarsening at later stages of phase separation for both 2D-RDP and 1D-LP may be studied via the interfacial energy $\sigma \equiv {\int }_{V}\,|\nabla \varphi {|}^{2}dV$. Figure 7(a) shows $\sigma$ over time for 2D-RDP when *h* = 8 and *V*_*xy*_ = 1000 and 1D-LP when *h* = 8 and *V*_*xy*_ = 0.1. We find that the interfacial energy in 2D-RDP is much lower than that in 1D-LP at later stages. When *h* = 8, we also find that $\sigma$ for 1D-LP remains constant at a high value for $t > 1000$ as shown in Fig. 7(a). This suggests that the 1D-LP is a stable structure over time, even though the pattern is energetically unfavorable. This is distinct from when *h* = 6, as shown in Fig. 7(b). At an early stage, $\sigma$ increases over time due to the formation of a 1D-LP like structure. This is followed by $\sigma$ drastically decreasing for $t > 1000$ as the 1D-LP structure transforms into 2D-CC pattern. This difference between *h* = 6 and *h* = 8 may be understood by considering the effect of curvature; a key point when considering the stability of a layer pattern is curvature, since coarsening is driven by the curvature of the interface^27^. When *h* = 6, the interface near the center is curved [see Fig. 5(b1)], leading to coarsening in the *z* direction. When *h* = 8, on the other hand, the interface is flat [see Fig. 5(c3)], making it difficult to overcome the energy barrier, consequently stabilizing the 1D-LP. This suggests that the percolation of the majority phase at an early stage is critical for allowing coarsening to transform a 1D-LP to a 2D-CC.

### Transition between 2D-CC and 2D-DP

**Figure 8:**
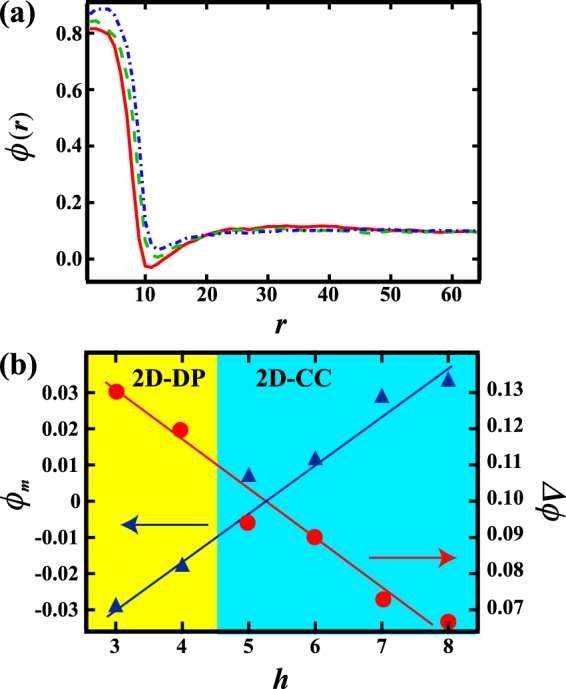
(**a**) Concentration profile in the radial direction $\varphi (r)$ as a function of distance from the center *r* at *z* = 0 and *t* = 150, when *V*_*xy*_ = 0.1. The solid line, dashed line and dot-dash line correspond to $\varphi (r)$ when *h* = 3, 5, and 8, respectively. (**b**) Minimum value ${\varphi }_{m}$ of $\varphi (r)$ and Δ$\varphi$ (=$\bar{\varphi }-{\varphi }_{m}$) at *t* = 150 as a function of *h*. ${\varphi }_{m}$ increases and Δ$\varphi$ decreases with increasing $h$. This suggests that coarsening in the azimuthal direction is slower for larger $h$. A 2D-DP is formed when $h\le 4$, while a 2D-CC is formed when $h\ge 5$.

Next, we consider the transition between 2D-CC and 2D-DP. The boundary between 2D-CC and 2D-DP shifts toward smaller ${V}_{xy}$ with increasing *h*. The transition between 2D-CC and 2D-DP is expected to be similar to the transition between a columnar and lamellar pattern during directional quenching^13,18^; just before the appearance of the first minority phase, a lower concentration region exists in the vicinity of the majority phase. This low concentration region aggregates in the *z* direction to lower the local free energy. When the aggregation velocity is faster than ${V}_{xy}$, the minority phase appears in the vicinity of the majority phase with a droplet shape, rather than a thin layer. Note that the aggregation velocity is related to $\Delta \varphi =\bar{\varphi }-{\varphi }_{m}$, where ${\varphi }_{m}$ is the concentration minimum in the low concentration region. When Δ$\varphi$ is large, the aggregation velocity becomes large. Here, we compute $\varphi (r)$ at *z* = 0 when *h* = 3, 5, and 8 and *V*_*xy*_ = 0.1, *t* = 150 as shown in Fig. 8(a). Although the temperature field on the bottom is the same, we find that the $\varphi (r)$ profiles depend on *h*. Flux at an early stage increases with increasing *h*, and there is a flux in the *z* direction to compensate. Thus ${\varphi }_{m}$ increases with increasing *h* and Δ$\varphi$ decreases as shown in Fig. 8(b). Considering the *h* dependence of Δ$\varphi$ as shown in Fig. 8(b), the aggregation velocity decreases for larger *h*. Since the boundary between 2D-CC and 2D-DP is determined by a balance between the aggregation velocity and ${V}_{xy}$, this results in a boundary shift toward smaller ${V}_{xy}$ with increasing $h$.

### Transition between 2D-RDP and 2D-CC

Finally, we focus on the transition between 2D-RDP and 2D-CC. Previous work using a two dimensional numerical simulation (*h* = 1) showed that the boundary is located at *V*_*xy*_ ~ 0.5^18^. Figure 1(a) shows that the boundary moves toward smaller ${V}_{xy}$ with increasing *h*; this is similar in nature to the boundary between 2D-CC and 2D-DP. Firstly, the majority phase emerges at the center at an early stage in both systems. In the vicinity of the majority phase, the concentration is lower than that of the mean concentration^27^. This low concentration region is subject to the influence of the radial quenching. If thermal fluctuations are larger than the influence of the radial quenching at the edge of the quenched region, phase separation may occur with the formation of random droplets. Note that the influence of radial quenching is larger for larger Δ$\varphi$. Figure 8(b) suggests that this influence is smaller for larger *h*. Thus, a 2D-RDP pattern appears when *V*_*xy*_ = 0.5 and *h* = 4 because thermal fluctuations are dominant near the edge of the quenched region, while a 2D-CC pattern is found when *h* = 3 for the same *V*_*xy*_. This is supported by an additional simulation without thermal noise; a 2D-RDP is not formed even at large *V*_*xy*_, and only 2D-CC patterns are found.

## Discussion

We consider the close analogy between the system simulated here and an experiment. In an actual experimental setup, the temperature is usually controlled by the action of a heating/cooling stage on a single side. Controlling the spatial temperature profile with high resolution using a heating/cooling stage is non-trivial. We thus consider how this might be realistically achieved in the laboratory. Take the use of illumination to control temperature. Firstly, we heat the sample by homogeneous illumination with light. We then locally shield the sample from the light with a barrier, the size and position of which may be regulated with a computer. The shielded region experiences a local lack of heat; thus, we may freely control the extent and position of a quenched region with excellent temporal and spatial resolutions. Preliminary experimental work in our lab has confirmed that this may be used to achieve a temperature quench from the center of the system, and that we may expand this region over time, but this is beyond the scope of this work.

## Summary

To summarize, we investigated the effect of confinement on radial quenching applied to the bottom surface of a three-dimensional box filled with a binary, phase separating system. We observed a range of patterns by changing $h$ and ${V}_{xy}$, including a 1D layer pattern, 2D random droplet pattern, 2D concentric circles, and 2D dendritic pattern. On top of factors known to influence homogeneous quenching e.g. the finite size effect and wetting, it was shown that the dominant flux in the system is changed from $z$ direction to the $xy$ direction by spatial confinement. This change in the flux direction strongly affects the concentration profile; a wide variety of patterns may be observed by changing the confinement $h$ in response. Anisotropic material flux can be ubiquitously observed in systems featuring temperature gradients, dissolution processes, evaporation etc. We hope that useful connections may be drawn from our interpretation of this system to explaining and control non-equilibrium phenomena for technological applications e.g. microfabrication in nanotechnology.

## Methods

Time evolution of phase separation with homogeneous quenching is often studied using the Cahn-Hilliard-Cook equation^27–29^. For an inhomogeneous temperature field, a modified Cahn-Hilliard-Cook equation was proposed^26^.

3 $$\begin{array}{rcl}\frac{\partial \varphi }{\partial t} & = & \nabla \cdot \{L\nabla [a(T(\overrightarrow{r})-{T}_{c})\varphi +b{\varphi }^{3}-c{\nabla }^{2}\varphi \\  &  & +\,\frac{J}{T}\nabla T\cdot \nabla \varphi -\frac{J}{4}\varphi (-\frac{1}{T}{\nabla }^{2}T+\frac{2}{{T}^{2}}{(\nabla T)}^{2})]-\overrightarrow{g}\}.\end{array}$$

where $\varphi$, $T$, $L$, $T$, and $\overrightarrow{g}$ are concentration, time, transport coefficient, temperature, and a random flux, respectively. $a$, $b$, $c$ and $J$ are positive constants. Assuming local equilibrium, $\overrightarrow{g}$ obeys the fluctuation-dissipation relation:

4 $$\langle {\overrightarrow{g}}_{i}(\overrightarrow{r},t){\overrightarrow{g}}_{j}(\overrightarrow{r^{\prime} },t)\rangle =2LT(\overrightarrow{r}){\delta }_{ij}\delta (\overrightarrow{r}-\overrightarrow{r^{\prime} })\delta (t-t^{\prime} ),$$

where $i,j=x,y,z$. The system studied here is initiated with a temperature $T(\overrightarrow{r})={T}_{0}(\, > \,{T}_{c})$ at all positions $\overrightarrow{r}$. ${T}_{c}$ is a critical temperature. We normalize the length and the time using the correlation length ${\xi }_{0}=\sqrt{c/a({T}_{0}-{T}_{c})}$ and the characteristic time ${\tau }_{0}={\xi }^{2}/La({T}_{0}-{T}_{c})$, respectively. We normalize the concentration $\varphi$ using ${\varphi }_{0}=\sqrt{a({T}_{0}-{T}_{c})/b}$. Next, we define a normalized temperature $\epsilon (\overrightarrow{r},t)$ as $\epsilon (\overrightarrow{r},t)=(T(\overrightarrow{r},t)-{T}_{c})/({T}_{0}-{T}_{c})$. We thus obtain normalized equations as shown below,

5 $$\begin{array}{rcl}\frac{\partial \varphi }{\partial t} & = & \nabla \cdot \{\nabla [\epsilon \varphi +{\varphi }^{3}-{\nabla }^{2}\varphi +\frac{J}{c}\frac{1}{T}\nabla T\cdot \nabla \varphi \\  &  & -\,\frac{J}{4c}\varphi (\,-\frac{1}{T}{\nabla }^{2}T+\frac{2}{{T}^{2}}{(\nabla T)}^{2})]-\overrightarrow{g}\}.\end{array}$$

The fluctuation-dissipation relation for the dimensionless noise is

6 $$\langle {\overrightarrow{g}}_{i}(\overrightarrow{r},t){\overrightarrow{g}}_{j}(\overrightarrow{r^{\prime} },t^{\prime} )\rangle =\Theta (\overrightarrow{r}){\delta }_{ij}\delta (\overrightarrow{r}-\overrightarrow{r^{\prime} })\delta (t-t^{\prime} )$$

7 $$\Theta (\overrightarrow{r})=\frac{T(\overrightarrow{r})b}{{c}^{3/2}{[a({T}_{0}-{T}_{c})]}^{1/2}}=\frac{{T}_{0}-\Delta T(\overrightarrow{r})}{{T}_{0}}\frac{{T}_{0}b}{{c}^{3/2}{[a({T}_{0}-{T}_{c})]}^{1/2}},$$

where Δ*T* is the quench depth from ${T}_{0}$. Here, we note that $J/c$ is almost the same as $\xi$^27^; thus, $J/c\nabla T\approx \Delta T$. When we perform a shallow quench, $\Delta T/T$ is quite small. For example, in a typical phase separation experiment, $T$ is ~300 K and $\Delta T\sim 10\,{\rm{K}}$. In such a case, the temperature gradient terms of Eq. (5) can be considered negligible. This is equivalent to saying that concentration transport due to the temperature gradient (Ludwig-Soret effect) is negligible^30^.

We noted above that the quenching was applied to the bottom surface, and that evolution of the temperature was governed by conduction. $\epsilon$ in the internal regions of the box may thus be computed using the thermal diffusion equation. Normalized equations are given below,

8 $$\frac{\partial \varphi }{\partial t}={\nabla }^{2}[\epsilon \varphi +{\varphi }^{3}-{\nabla }^{2}\varphi ]+\theta $$

9 $$\frac{\partial \epsilon }{\partial t}=Le{\nabla }^{2}\epsilon ,$$

where $\theta =-\,\nabla \cdot \overrightarrow{g}$. $Le(\,=\,K/L)$ is the Lewis number, where $K$ is a thermal diffusion constant. When $\epsilon$ is positive, the mixed state is stable; when $\epsilon$ is negative, phase separation occurs. We solved these partial differential equations using the Euler method. We set the mesh size to 1 and the time step to 0.01. The simulation box size was *x* : *y* : *z* = 128 : 128 : *h*; *h* is a positive integer and the key parameter here for investigating the effect of confinement. Note that we use a staggered grid for this simulation. The bottom and the top surfaces of the simulation box are located at *z* = −1/2 and $h-1/2$, respectively. The temperature of the bottom surface *z* = −1/2 in the simulation is defined by

$$\epsilon (x,y,-\,1/2,t)=\{\begin{array}{ll}1 & |{\overrightarrow{r}}_{xy}-{\overrightarrow{r}}_{c}| > \sigma (t)\\ -1 & |{\overrightarrow{r}}_{xy}-{\overrightarrow{r}}_{c}| < \sigma (t)\end{array}$$

where ${\overrightarrow{r}}_{xy}$ and ${\overrightarrow{r}}_{c}$ are position vectors corresponding to positions on the bottom surface and of the center of the radial quenching, respectively. $\sigma (t)$ is the distance from the center to the front of the quenching region at time *t*. Then, the temperature at each point is defined as $\epsilon (x,y,z)=[\epsilon (x,y,z+1/2)+\epsilon (x,y,z-1/2)]/2$. We also set a boundary condition for the top surface, given by

$${\frac{\partial \epsilon }{\partial z}|}_{z=h-1/2}=0.$$

This denotes that the top surface is thermally insulated. A boundary condition is also defined for the concentration as

$${\frac{\partial \varphi }{\partial z}|}_{z=0,h-1}=0.$$

This implies that the two surfaces are perfectly neutral i.e. no surface field acts on the mixture, unlike the problem of surface-directed spinodal decomposition^8–11,31–33^. We used periodic boundary conditions in the $xy$ plane. For simplicity, $K$ and $L$ were set as constant, and we set *Le* = 100. *Le* in realistic materials is much larger than 100; however *Le* = 100 is large enough since the temperature has quickly reached equilibrium before phase separation occurs. Then $\Theta (\overrightarrow{r})$ is set as constant since we neglect the term $\Delta T/{T}_{0}$. We set $\Theta (\overrightarrow{r})=0.001$ at any $\overrightarrow{r}$, which corresponds to a noise amplitude at $T(\overrightarrow{r})={T}_{0}$. The mean concentration is set to $\bar{\varphi }$ = 0.1; note that this denotes an asymmetric composition. Having set up the simulation in this manner, we start radially quenching outwards from a point on the bottom surface with a constant velocity ${V}_{xy}$ at $t=0$ i.e. $\sigma (t)={V}_{xy}t$.
